# The Triple P Positive Parenting Program for Parents With Psychosis: A Case Series With Qualitative Evaluation

**DOI:** 10.3389/fpsyt.2022.791294

**Published:** 2022-02-22

**Authors:** Lauren Wolfenden, Rachel Calam, Richard J. Drake, Lynsey Gregg

**Affiliations:** ^1^Division of Psychology and Mental Health, School of Health Sciences, Faculty of Biology, Medicine and Health, Manchester Academic Health Science Centre, The University of Manchester, Manchester, United Kingdom; ^2^Greater Manchester Mental Health NHS Foundation Trust, Manchester, United Kingdom

**Keywords:** parental mental health, parenting, schizophrenia, SMI, parent-child interaction

## Abstract

Although many people with psychosis are parents, managing the dual demands of poor mental health and parenting can be stressful and may contribute to poorer outcomes for both parent and child. Parenting interventions have the potential to improve outcomes for the whole family but need evaluation of feasibility in this context. The Triple-P Self-Help Workbook was implemented with guidance and support with 10 parents experiencing psychosis in a multiple baseline case series study. Sessions were weekly and home-based. Outcome measures examined facets of parenting, child behavior, self-efficacy and parental mental health. Follow up interviews explored parents' perspectives of the perceived impact of the intervention and apparent mechanisms of change. The program resulted in clinically significant change (>25% improvement) in mental health, parenting and child behavior measures post-intervention for the 50% who completed all 10 sessions and improvements were maintained at 3 and 6 month follow up. Interviews with those who completed the program revealed it to have been transformative: parents reported positive changes in parenting style; they were empowered with regard to their parenting and had a greater sense of control over their mental health. This study provides preliminary evidence that self-directed Triple P might be able to reduce the symptoms of psychosis by improving family functioning. Findings could inform the future development or adaptation of evidence-based parenting interventions for parents with psychosis in order to improve their mental health, aid recovery, and intervene early in the lives of children at risk of poor long-term outcomes.

## Introduction

Serious mental illness (SMI), such as psychosis, can be debilitating and interfere with social, emotional and psychological functioning. The chronicity and severity of symptoms experienced in psychosis can have multifaceted and debilitating implications for daily life (1). Without support, the impact on mood, relationships and quality of life can be profound (2). Such challenges can be further exacerbated when people with psychosis also have dependent children with elevated emotional reactivity to stress, making parenting a particularly stressful aspect of their lives. At the same time, the parental role can create meaning, belonging and increase self-worth and as such, is an important part of self-identity for both men and women with psychosis (3, 4) and may be an important focus for recovery (5, 6).

It has been estimated that up to 55% of men and 62% of women experiencing psychosis are parents (7) but current treatments may neglect the challenges experienced by these parents, particularly mothers (4).

The link between parental SMI and reduced quality parent-child interactions, poor attachments and limited sensitivity is well established (8–11) and the influence of parental factors in the development, maintenance and amelioration of disruptive behaviors and psychological health of children has been evidenced within practice and comprehensive reviews (12–16).

However, experiencing SMI does not need to impede the ability to parent effectively (17, 18); interventions that target parenting and child behaviors and parental mental health have potential to prevent long-term adverse consequences for families and should be prioritized (14, 19–21). The use of evidence-based parenting interventions is recommended by the National Institute for Health and Clinical Excellence to improve child behavioral problems and reduce intergenerational cycles of poor mental health (22) however, there remains a lack of appropriate and timely parenting interventions to support parents experiencing SMI, and in particular, psychosis.

One widely used parenting intervention is the Triple P-Positive Parenting Program (23, 24). Based on social learning theory and cognitive behavioral principles, it aims to improve confidence in the parenting role and modify maladaptive parenting behaviors (25). The self-directed variant also targets coercive family interactions and offers skill acquisition and problem solving (26, 27). Large scale trials of the self-help variant have demonstrated positive outcomes for both parents and children similar to that of standard parenting interventions (24, 28, 29) including with parents experiencing bipolar disorder (30, 31).

To date, no study has evaluated the use of self-help Triple P with parents experiencing psychosis. The aim of the current study was to ascertain whether the use of this intervention in parents' homes was feasible and acceptable and whether there were any clinical effects for parents or children during and upon completion of the intervention in terms of child behavior and parental mental health. A single case design with multiple participants was used in order to capture the impact of the intervention on each individual participant rather than obtain an aggregate group effect which was not concerned with individual experience.

## Methods

### Design

A within-subject A-B-A single case design across participants with follow-up was implemented. In this design, which employs a multiple baseline phase, participants act as their own controls. Following the baseline period (A), the ten sessions of the intervention (B) were delivered weekly over 10–14 weeks. After the intervention, participants were followed up at 3 and 6 months (A). The intervention was initiated only if the repeated measurements at baseline were stable, or else the baseline phase was increased. The repeated assessment of dependent variables during all three phases allows for the dependent variables to be assessed prior the implementation of the intervention and confidence is increased that any observable changes are attributable to the intervention rather than alternative explanations.

Qualitative methodology was employed to generate knowledge surrounding implementation, usefulness and perceived change based on parents' personal accounts of engaging in the program. Study procedures were registered prior to recruitment (clincaltrials.gov: NCT02622048).

### Participants

Participants were eligible to take part if they met the following criteria: (1) ability to provide informed consent; (2) English speaking; (3) diagnosed with Schizophrenia Spectrum Disorder; (4) parent of a child aged 3–10 years old with whom they had more than 10 h of face to face contact per week; (5) over 18 years old; (6) medication stable; and (7) no change in care plans and no other parenting support being received. Case note review corroborated diagnosis or symptoms and was additionally considered by a psychiatrist (author 3) using the International Classification of Diseases (ICD) version 10 (32). Participants were referred from Early Intervention Services (EISs), Community Mental Health Teams (CMHTs) and local authority services, including local council family and housing teams.

### Data Collection and Evaluation

Data were collected for parents who took part in the intervention to monitor feasibility, change over time and acceptability. Delivery adaptations were also recorded. In line with process evaluation planning (33–35), 11 key areas were highlighted as priorities: recruitment, maintenance, context, resources, implementation, reach, barriers, exposure, initial use, continued use and contamination.

Primary outcomes included attrition rate monitoring, proportion of data points completed and acceptability. In line with the MRC Framework, mechanisms of impact were explored using the client satisfaction questionnaire to provide a quantified measure of perceived acceptability, usefulness and practicality. Qualitative interviews were also undertaken following completion of the intervention. Secondary outcome measures consisted of weekly (continuous) measurement of symptoms, mood, parental efficacy and child behaviors. Additional outcome measures assessed social functioning, parenting practices and family relationships at the start and end of baseline phases and at the end of the intervention. During such preliminary research, incorporating a range of outcome measures at different time points can help to ascertain how participants interpret each measure and understand perceived usefulness.

### Measures

Well-established and validated semi-structured interview schedules were employed to assess symptoms and functioning: The Positive and Negative Syndrome Scale [PANSS; (36)], Psychotic Symptoms Rating Scale [PSYRATS; (37)] and the Personal and Social Performance Scale [PSP; (38)] were used to assess psychopathology and functioning. The self-report Depression, Anxiety and Stress Short Form Scale [DASS-21; (39)] was used to determine parental mood and stress levels. Parenting and child behaviors were explored using a range of measures: the Parenting Task Checklist [PTC, (40)] assessed parental self-efficacy, the Eyberg Child behavior Inventory [ECBI, (41)] assessed intensity and frequency of problematic child behaviors and the Parenting Scale [PS, (42)] assessed a range of parenting behaviors. Additionally, the Family Background Questionnaire [FBQ, (43)] was used to collect demographic and psychosocial information. The Client Satisfaction Questionnaire [CSQ; (24)] assessed parents' thoughts and beliefs regarding the acceptability and effectiveness of the parenting intervention for themselves, their family and their child.

### Procedure

Following referral from the healthcare professionals working with parents, parents who met the inclusion criteria were given a written participant information sheet (PIS) to read before participating in an initial visit with the researcher. At this visit study processes and the nature of the work book were explained and discussed. If literacy problems were noted, the researcher read the PIS aloud before giving parents the opportunity to ask questions. At a second visit, at least 48 h after the first, informed consent was obtained. Participants with more than one child were asked to identify a target child with whom they experienced the most difficulties. Data were recorded for this child only.

Participants were monitored and assessed using a multiple baseline approach (A), during weekly home-visits over the 10-week intervention (B), repeat of baseline (A) and at three-and 6-month follow-up. The first baseline phase acted as an engagement opportunity and built rapport with participants; this ensured safety and trust, and facilitated commitment to the program. Following the engagement phase, the intervention began and weekly symptom monitoring occurred. Changes to mental health, current parenting behaviors and child behavior were measured using the PANSS, DASS-21, ECBI and PTC. In addition to measures used during the pre-and post-intervention multiple baseline phases (A) the PSYRATS, PSP and the PS were used during weekly monitoring.

Typically, baseline visits lasted 45–60 min over a minimum of three sessions. The baseline phase controlled for potential confounds, ensuring any change could more likely be attributed to participation in the program (44, 45). The intervention phase consisted of a minimum of 10 weekly visits lasting 1.5 h.

Follow up interviews took place in participants' homes, where a flexible interview schedule consisting of open-ended questions was used to explore experiences of the program in two broad domains: (i) experiences of taking part in the program in relation to self, child and parent-child relationship; and (ii) perceived intervention appropriateness and effectiveness. Interviews were audio recorded and transcribed verbatim. Duration varied between 46–57 min (mean = 52 min).

### The Triple P Positive Parenting Program

The self-directed variant of the Triple P Positive Parenting Program was used in its manualized “Every Parent”s Self-Help Workbook' format. The workbook aims to promote self-sufficiency and independence to achieve sustainable behavior change over 10 weeks. The work book focusses on 17 core strategies; 10 of which are designed to promote child development (i.e., attention through quality time and talking; affection; praise; engaging activities; incidental teaching; parent as role model; and daily/weekly behavior monitoring charts). The final seven encourage parents to actively manage misbehavior (i.e., appropriate rule setting and boundaries; directed discussion and instructions; logical consequences, quiet-time and time-out) (26). The skills acquired aim to help parents to form clear plans that can be used at home and in the community to respond well in situations that become difficult or “high risk”.

Sessional role plays to practice learnt techniques were used to reinforce the development of self-evaluation and problem-solving capabilities. Parents were aided to explore and understand their child's needs and causes of behavior, emotional and social problems. Such tasks aimed to enable participants to acquire skills to aid their awareness of appropriate expectations and child development. These were deemed low level adaptations that did not alter the intervention (46). All sessions and assessments occurred in the client's home and were conducted by the first author.

Due to complex family circumstances and readiness levels, a flexible, parent-led approach was used throughout. Additional support was offered in the form of (a) help reading the workbook, ensuring parents understood the content and examples used and (b) conversations linking mental health to parenting. This facilitated engagement, problem solving, and implementation of strategies within the workbook. The conversations linking mental health to parenting helped participants who were not already doing so make links between their parenting and mental health. These conversations took place after the outcome measures were assessed each week. Participant's responses to the self-reported mental health, parenting and child behavior measures were discussed in relation to the strategies in the workbook the parent was attempting to develop. In this way, parents were encouraged to begin to recognize their triggers, their child's triggers and also their strengths rather than deficits.

Potential facilitators and barriers to implementation were identified within baseline sessions. In this phase parenting strengths, struggles and areas of desired change were identified and fed into the intervention to ensure goals were individual to participants' needs. Participants were sent text messages the day before and the day of each session to remind them of the upcoming session and ensure tasks had been completed beforehand.

#### Contextual Factors: Guided Self-Help Adaptations

There were high rates of challenging circumstances for each family, such as: literacy problems, family conflicts and disruptions, social isolation, fear, poverty and financial stressors. Therefore, during the initial baseline sessions time was spent collaboratively identifying parents' strengths and struggles. More than half experienced literacy problems (60%), some reported that they had never actively read a book before (30%), and most had never read for pleasure (80%). It was therefore essential to make minor adaptations to the delivery of the self-directed workbook and time spent completing practical exercises. Participants required assistance to understand tasks and required support and guidance when planning and implementing the strategies. This was deemed “low risk” because it did not change the core elements of the intervention or the measures used (47).

### Data Analysis

SPSS 25.0 was used for all statistical analyses. Visual inspection of graphical data, percentage change calculations of outcome measures and descriptive statistics were used to ascertain the acceptability and feasibility of the intervention. Means were derived from measurements at each time point. Data were graphically represented for weekly measurements to explore change over time. There are numerous established ways to calculate clinical significance which produce similar outcomes (48). We used the Reliable Change Index approach to calculate clinically significant change (49). Clinically significant change was defined as > 25% reduction from pre to post intervention (50). A threshold of 25% change was classified as a “moderate outcome” and > 50% classified as a “good outcome” based on previous studies using the PANSS and PSYRATS (51, 52). For participants who elected to finish using the workbook before session 10, weekly scores to that point were used to monitor change.

End of program interview transcripts were analyzed using a framework analysis approach (53) in which the interview topic guide was used to provide an initial framework for the analysis. Coding was also inductive allowing for the expansion or collapsing of initial themes and categories before production of the final classification framework. To preserve anonymity participants' names were replaced with a pseudonym and names of children were replaced with “X”.

## Results

### Participant Characteristics

All participants were experiencing psychosis and were mothers of at least one child aged between 3–10 years old. Table 1 provides a breakdown of participant characteristics and family circumstances.

**Table 1:** Participant and family characteristics.

	**Parent details**							**Target child details**	**Completed programme**
	**Sex**	**Age**	**Diagnosis**	**Ethnicity**	**Marital status**	**Employment**	**Number of children**		
Participant 1	Female	33	Schizophrenia	White British	Single	Unemployed	3	Male, 7	No
Participant 2	Female	36	Schizophrenia	White British	Single	Unemployed	3	Female, 10	No
Participant 3	Female	26	Schizophrenia	White British	Single	Unemployed	4	Male, 10	No
Participant 4	Female	48	Schizophrenia	White British	Single	Unemployed	2	Male, 9	Yes
Participant 5	Female	25	Schizophrenia	White British	Single	Unemployed	2	Male, 4	Yes
Participant 6	Female	28	Schizophrenia	Black African	Single	Unemployed	2	Male, 9	Yes
Participant 7	Female	33	Schizophrenia	White British	Single	Working part time	2	Female, 8	No
Participant 8	Female	27	Schizophrenia	White British	Single	Unemployed	2	Male, 6	Yes
Participant 9	Female	40	Schizophrenia	White British	Cohabiting	Unemployed	5	Female, 8	Yes
Participant 10	Female	33	Schizophrenia	Chinese	Single	Unemployed	1	Male, 9	No

Participants were white British (8, 80%), Black African (1, 10%) and Chinese (1, 10%). They were mainly single parents (9, 90%) who had a diagnosis of schizophrenia (6, 60%) or paranoid schizophrenia (4, 40%). One participant had completed higher education (10%), qualifications were otherwise at high school (2, 20%) or sixth-form level (7, 70%). The majority (90%) were unemployed and all reported that they were struggling financially with either “just enough to meet essential expenses only” (3, 30%) or “unable to meet essential expenses” (7, 70%).

### Feasibility

#### Enrolment

**Figure 1:**
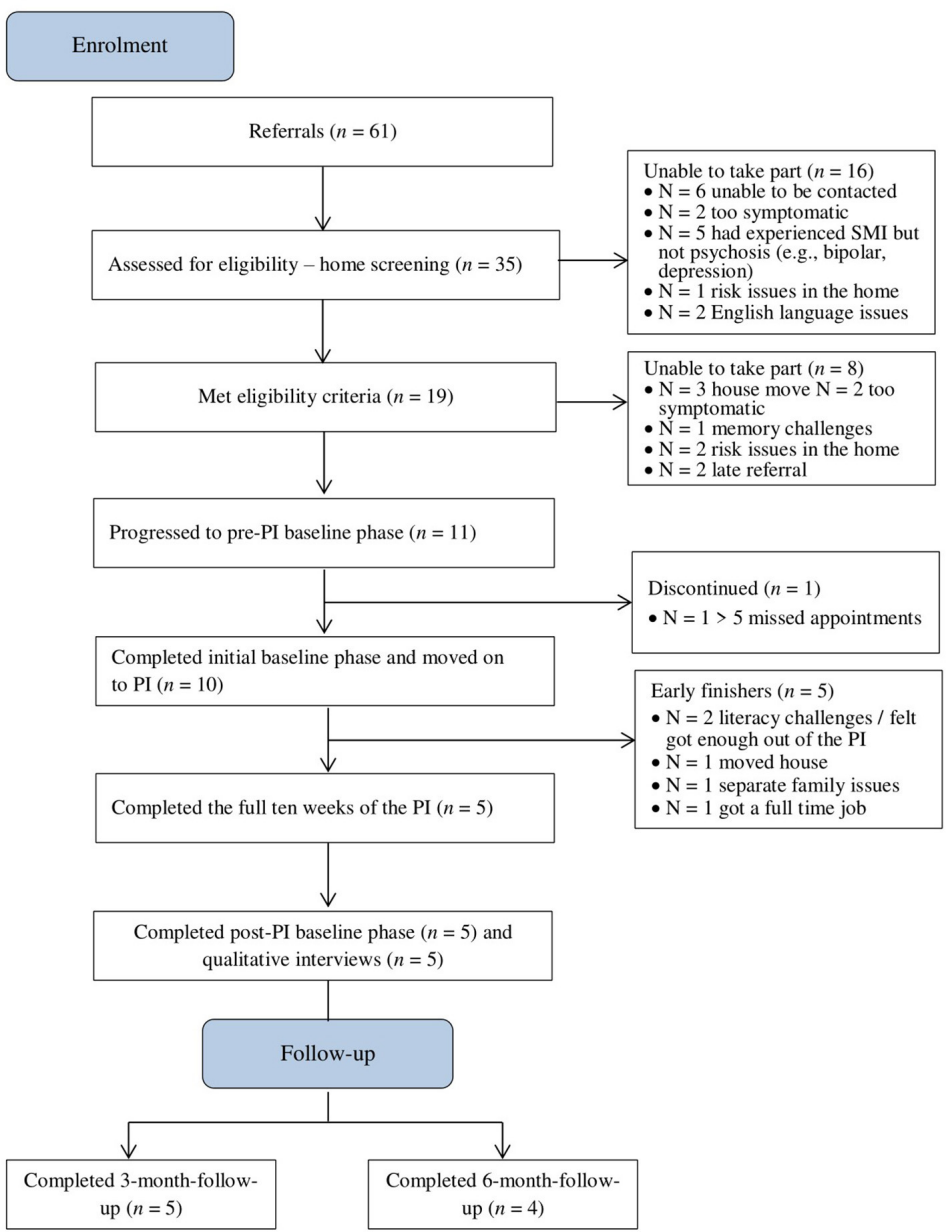
CONSORT diagram showing participant flow through the study.

Recruitment was successful: a total of 19 parents referred to the study were eligible to take part and all expressed an interest in participating, however only 11 progressed to the initial baseline phase. No participants declined participation. The CONSORT diagram is presented in Figure 1.

#### Attendance and Attrition Rates: Uptake of Intervention

The maximum number of visits from baseline to follow-up was 23 per participant, 230 in total across the sample. Of these 171 (74%) were attended. Four participants attended all possible sessions, one attended 91%, three attended 52% and two attended 48%.

Of the 11 parents who progressed to the initial baseline phase one did not complete baseline due to more than five missed appointments. Ten completed the baseline phase and progressed to the intervention. Of these, five completed all 10 weeks of the workbook, final baseline phase and 3-month-follow-up. Four of these completed the 6-month-follow-up. The remaining five partially completed the workbook, completing to either week 4 or week 6. The five participants who did not complete it discontinued for practical reasons (a house move; new employment and challenges with another child in the family, *N* = 3) or because they found the workbook challenging due to poor literacy (*N* = 2).

All parents completed the first half of the workbook which contains the core strategies which promote positive parenting practices, self-monitoring strategies, stress reduction, and management of disruptive child behaviors. The five parents who finished early failed to cover troubleshooting, practice strategies, and identifying future challenges in high risk situations. No adverse events, hospital admissions or worsening of symptoms were reported post-intervention or at follow-up visits.

### Acceptability Ratings

Overall, the intervention was rated as highly acceptable by eight participants with an average score of 95% (range: 76–100%) on the client satisfaction questionnaire. It was rated as 87% (range 71–100%) “Useful and informative”, 89% (range 71–100%) “Interesting” and 79% (range 57–100%) “Practical”.

### Clinical Outcomes

**Figure 2:**
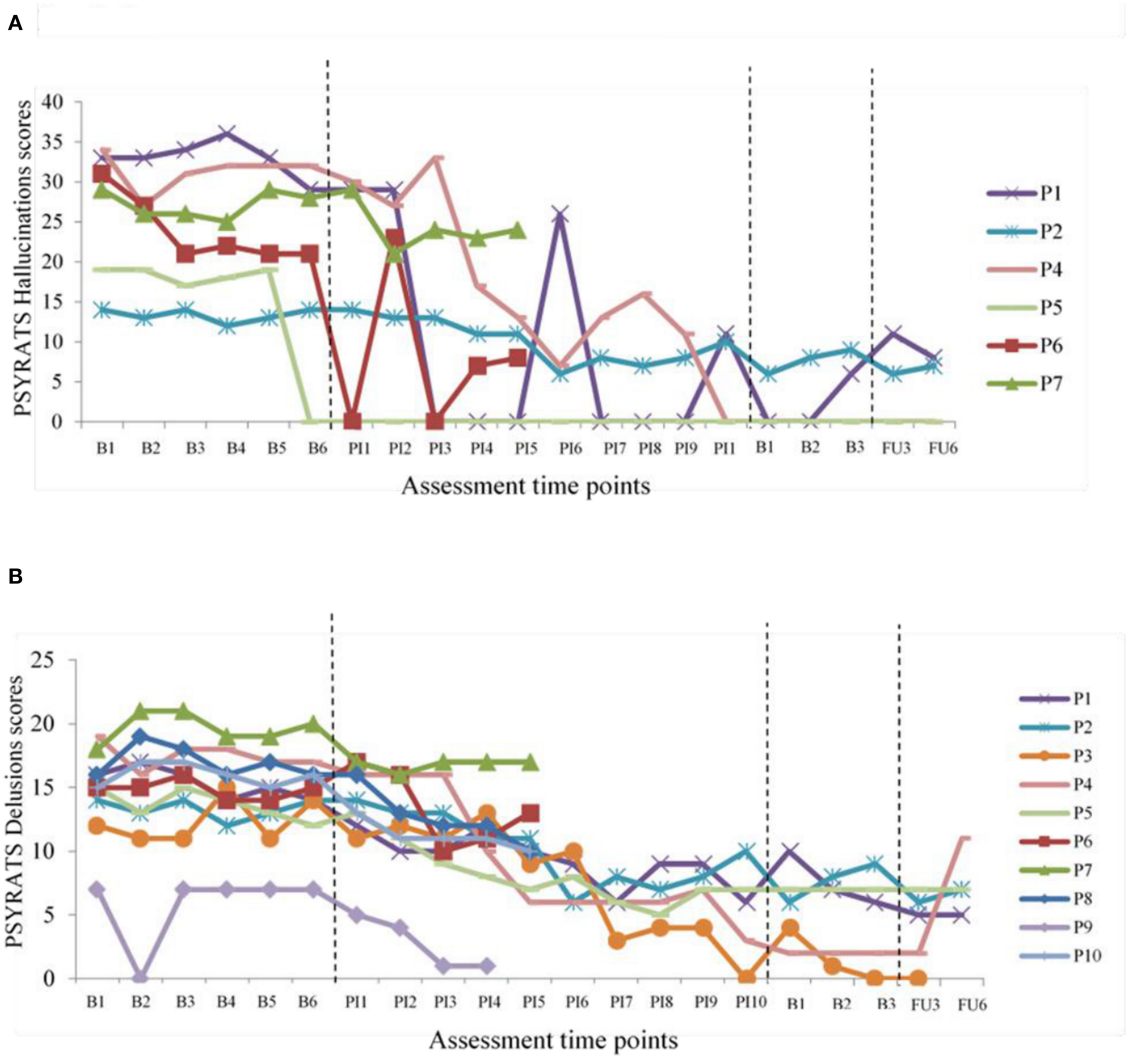
Changes in PSYRATS Hallucinations and Delusions. **(A)** PSYRATS Hallucination scores over time. **(B)** PSYRATS Delusions scores over time.

**Figure 3:**
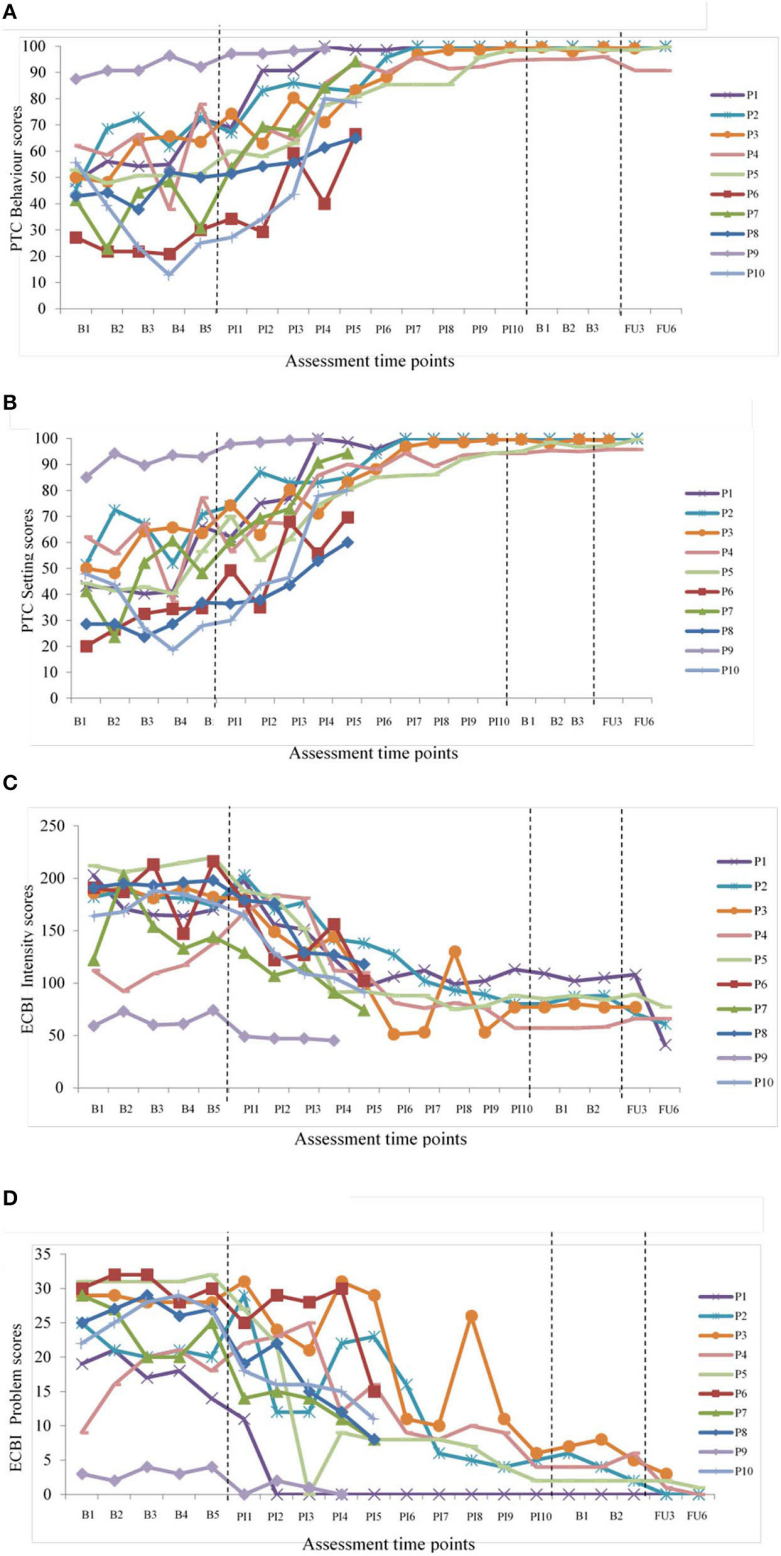
Changes in parenting and child behavior. **(A)** PTC behavior scores over time. **(B)** PTC setting scores over time. **(C)** ECBI intensity scores over time and **(D)** ECBI problem scores over time as per the file in the original submission.

Analyses of the effects of taking part in the intervention on mental health and parenting measures are summarized in Table 2. Tests of significance (*t*-tests) and effect sizes (Cohen's d) are presented. Graphical representations of weekly mental health outcomes can be found in Figures 2A,B, and weekly parenting and parent-child outcomes can be found in Figures 3A–D.

**Table 2:** Pre and post intervention measures.

**Measures**	**Pre PI baseline mean (SD)**	**End of PI mean (SD)**	**Post PI baseline mean (SD)**	**Follow-up 3 month (SD)**	**Follow-up 6 month (SD)**	**Pre to post PI**			**Pre to follow-up 3 months**			**Pre to follow-up 6 months**		
	**(*n* = 10)**	**(*n* = 5)**	**(*n* = 5)**	**(*n* = 5)**	**(*n* = 4)**	***t***	***p***	***d***	***t***	***p***	***d***	***t***	***p***	***d***
**Parenting and child behavior measures**														
PTC behavior	49.7 (18.78)	89.2 (14.33)	98.26 (2.21)	98.4 (1.88)	98.84 (2.1)	9.5	0.001	2.49	12.1	0.001	4.08	−8.9	0.003	4.24
PTC setting	52 (22.96)	89.6 (14.1)	98.62 (1.9)	97.7 (3.9)	97.6 (3.9)	8.2	0.001	2.09	12.8	0.001	3.1	9.6	0.002	3.19
PS total	4.06 (0.77)	2.33 (0.622)	1.75 (0.81)	2.15 (0.45)	2.01 (0.67)	5.88	0.004	2.76	10.12	0.001	3.39	8.9	0.003	3.28
ECBI intensity	69.4 (12.6)	46.6 (6.7)	46 (4.7)	45.8 (4.6)	43.2 (3.6)	8.5	0.001	2.38	6.04	0.003	2.78	5	0.01	3.26
ECBI problem	70.3 (10.6)	47.6 (6.55)	45.2 (3.4)	42.4 (1.67)	41.2 (0.5)	7.5	0.001	2.71	8.9	0.001	4.11	7.11	0.005	4.47
**Mental health measures**														
PSYRATS hallucination	16.1 (14.7)	6.3 (9.2)	3.81 (7.01)	5.4 (7.6)	5.3 (6.7)	2.79	0.021	0.82	3.23	0.032	0.96	5.78	0.010	1.0
PSYRATS delusions	14.5 (3.8)	7.8 (5.32)	5.03 (3.01)	4.0 (2.91)	7.65 (2.3)	5.30	0.000	1.20	6.45	0.003	3.13	9.02	0.003	2.24
DASS-21 total	81.7 (35.26)	38 (32.37)	30.5 (33.4)	32 (36.9)	38 (41.6)	4.13	0.003	1.36	2.3	0.083	1.54	1.5	0.230	1.26
WEMWBS	31 (2.89)	52 (6.16)	47 (16)	53 (9.08)	57 (8.04)	6.16	0.004	4.88	4.5	0.011	3.65	5.5	0.012	5.17
PSP	46.8 (6.68)	62.4 (2.4)	63.2 (4.86)	63.4 (4.7)	65.5 (4.1)	5.24	0.006	3.43	6.12	0.004	2.91	4.52	0.020	3.46
PANSS positive	18 (5.6)	14.6 (3.78)	12.2 (4.54)	13.4 (4.39)	14.5 (3.87)	1.14	0.319	0.72	1.65	0.174	0.92	1.13	0.34	0.74
PANSS negative	13.4 (2.04)	10.6 (0.89)	9.2 (2.86)	10.2 (1.78)	10.0 (2.7)	2.44	0.071	1.9	2.02	0.114	1.68	1.43	0.247	1.43
PANSS general	33.5 (9.28)	26.6 (2.96)	23.4 (6.9)	25.6 (2.9)	25.5 (3.3)	1.59	0.186	1.13	1.99	0.117	1.3	1.73	0.182	1.27
PANSS total	64.8 (15.7)	51.8 (6.6)	44.8 (13.4)	49.2 (8.3)	50 (7.4)	1.61	0.182	1.16	2.01	0.115	1.3	1.50	0.230	1.28

#### Parental Mental Health

All participants experienced some reductions in symptom severity according to the PANSS (positive, negative and general subscales and overall total). This change was not statistically significant on the PANSS subscales but four of the five participants who completed 100% of the 10 week program achieved clinically significant reductions in PANSS total scores. At post-intervention symptoms were reduced by 25–44% from baseline for all participants. At the three-month-follow-up, clinically significant reductions were reported for all participants from baseline ranged from 29 to 39% and from 36 to 45% at 6 month-follow-up.

There were statistically significant improvements in both the hallucinations and delusions dimensions according to the PSYRATS. These improvements were maintained at both follow-up time points. Clinically significant change of >25% was observed for five of the six participants experiencing hallucinations and 9 of 10 participants experiencing delusions. For hallucinations, reduction in symptoms ranged from 66 to 100% post-intervention compared to baseline, from 47 to 100% at 3-month-follow-up and from 53 to 100% at 6-month-follow-up. For delusions, change ranged from 25 to 100% post-intervention compared to baseline, from 49 to 100% at 3-month-follow-up and 37–64% at 6-month-follow-up. Participants 1, 2, 4, 5 and 6 showed significant reductions in hallucinations frequency, severity and levels of distress. Participant three was no longer experiencing hallucinations or delusions. Graphical representations can be seen in Figure 2.

There were significant improvements in depression, anxiety and stress (DASS-21) pre to post intervention. However, this change was not maintained at follow-up. At baseline most participants fell into “severe” or “extremely severe” categories for depression (60%), anxiety (80%) and stress (80%). All scores improved and moved into “normal”, “mild” and “moderate” categories at completion, with 20–40% scoring zero across subscales. Percentage change improvements of >25% were observed for nine out of 10 participants on subscale and total scores at all time points. Change ranged from 27 to 100% post-intervention or final session (chosen end point) compared to baseline, and from 55 to 86% at follow-up. Graphical representations can be seen in Figure 2.

There were significant improvements in social functioning (PSP) post-intervention. These changes were maintained at both follow-up time points. At baseline, eight out of 10 participants were categorized as having “marked” or “severe” poor social functioning and two out of 10 into “marked” or “manifest” difficulties. Social functioning scores showed improvements at all-time points for all participants. Social functioning improved post-intervention and categorized participants as “mild” and “manifest”. Percentage change improvements of >25% was observed for three out of five participants who completed the program at all-time points. Changes ranged from 27 to 63% post-intervention compared to baseline, from 25 to 63% at 3-month-follow-up, and 35–78% at 6-month-follow-up. Participant 3 and 5 showed small improvements across all time points. This suggests improvements across facets of relationships, activities, self-care and aggressive behaviors.

#### Parenting

There were significant improvements in parenting on all PS subscales and total scores. These improvements were maintained at both follow-up time points. At baseline, eight out of 10 participants were within the clinical range expressing difficulties in their parenting role. Following the intervention, all participants fell below the clinical cut-off (3.2) and were within the normal range for parenting difficulties. Percentage change improvements >25% were reported for all five participants on PS total post-intervention compared to pre-intervention and at follow-up, and subscale improvements ranged from 18to 68%. PS total score improvements ranged from 28 to 54% post-intervention compared to baseline, from 41 to 53% at 3-month-follow-up and 40 to 58% at 6-month-follow-up.

Parental confidence also improved according to PTC behavior and setting subscales. These improvements were maintained at both follow-up time points. For PTC behavior subscale, improvements ranged from 58 to 142% post-intervention compared to baseline, from 59 to 115% at 3-month-follow-up and 59–121% at 6-month-follow-up. For PTC setting subscale, improvements ranged from 43 to 173% post-intervention compared to baseline, 50–94% at 3-month-follow-up and 55–97% at 6-month-follow-up. These improvements demonstrated change in parental self-efficacy in most settings and when dealing with most child behaviors following the intervention. Graphical representations can be seen in Figure 3.

#### Child Behavior

There were significant improvements in child behavior according to ECBI intensity and problem subscales. These improvements were maintained at both follow-up time points. At baseline, eight out of 10 participants fell into the ‘clinical' range for intensity scores (>131) and nine out of 10 on problem scores (>15). Following the intervention, participants who completed T1 and T2 were no longer scoring in the clinical category for child behavior problems or parental distress. Child behavior improvements of >25% was observed for nine out of 10 participants on the intensity subscale and all participants on the problem subscale post-intervention or chosen end point compared to baseline. For ECBI-intensity subscale, child behavior improvements ranged from 31%-59% post-intervention compared to baseline, from 38 to 61% at 3-month-follow-up and 42–55% at 6-month-follow-up. For ECBI-problem subscale, child behavior improvements ranged from 58 to 100% post-intervention compared to baseline, from 90 to 100% at 3-month-follow-up and 97–100% at 6-month-follow-up. Graphical representations can be seen in Figure 3.

### End of Study Qualitative Evaluation

The qualitative interviews aimed to uncover parents' experiences of the program, identifying aspects they found valuable or difficult in order to inform future implementation. In an attempt to uncover mechanisms of change we asked parents to reflect on their experiences of parenting prior to starting the program, and describe any positive impacts during and since its completion. The analytical framework organized the data into two main themes: The first theme describes the family's journey (parenting prior to the program, their expectations of the program and finally, program outcomes) and the second theme details the aspects of the program they found most valuable and any suggestions parents made for adaptations that they felt might be beneficial for other parents in in the future.

#### Theme 1: The Family's Journey

##### Parenting Prior to the Program

Parents described themselves variously as “hopeless”, “lost” and a “bad parent” before the program. One parent went as far as to say “I wasn't a Mum”. All five felt they were failing in the parental role and reported poor relationships with their children. Parental accounts highlighted a lack of control. They described cycles of behavior in which children's behavior was exacerbated via conflict between the parent and child, with “screaming and shouting” the norm. Attempts at control were sometimes futile: “everything I tried to do backfired in my face” (participant 9) and parents sometimes chose the “easy option” of not responding to challenging behavior and giving in “for a quiet life” despite recognizing that this could serve to worsen behavior.

“that's probably resulted in his difficult behavior cause he knows that it's all right, I'll just kick off get emotional and my mum will just end up getting it me, she won't follow through with it” (participant 6)

It was clear that self-efficacy for all five parents was low and this was also reflected in the quality of parent-child interactions. Parents described dreading spending time with their children and reported a desire to escape:

“I used to dread picking him up from school. I used to dread just being on my own with him… I'd say to my parents please let him come and stay at yours” (participant 4).

Significantly, although all were asked, no parents were able to identify anything positive about their parenting prior to the program and this question elicited an emotional response for some.

Parents reported their children's behavior to have been very problematic, describing disruptive and sometimes destructive behavior that drew comments from others, including wider family members and school teachers. This sometimes led to parents feeling judged and embarrassed and in some cases made them reluctant to be out in public with their child: “I was scared of being alone with him, I was worried if I had to take him out in how you say it, a public situation” (participant 4).

Parents noted the interplay between their children's behavior, their own mental health and parenting before the program, revealing very stressful family environments:

“I think probably my mental health and the fact I was always moody and miserable and shouting was obviously just fueling the fire… it just meant that he could play up even more and he could be as destructive as he wanted and stubborn as he wanted because I was not able because of my mental health to deal with it” (participant 4).

“…just all the stress, all the worries, all the screaming, all the fighting, all the shouting probably kept me up at night, so that's probably what caused the mental illness more than anything else really, just not being able to know how to deal with things” (participant 8)

“there was no rules, there was no instructions, there was no backup plans, there was no charts for doing good behaviors, there was no praise, there was no organization, it was chaos” (participant 8).

##### Expectations at the Start of the Program

Feelings of hopelessness prior to the program were echoed in low expectations about what the program could achieve. The two parents who had previously completed parenting courses reported not to have derived any benefit and expectations were again low: “when I was first told about this one I was like oh no not again” (participant 9). Parents who had not previously attended parenting courses were no more optimistic:

“I kind of felt like it wouldn't work at the start erm, just reading through it was kind of like, how did it feel like, how, can't believe I'm doing this… I'm going to bear to do this but in my heart of hearts I feel like this is totally not going to work” (Participant 6)

Another had not felt that she needed support despite struggling with her children “because I didn't think that there was a special parenting way” (Participant 8)

These misgivings were echoed in participant's preliminary perceptions of the workbook. Initial views were negative or neutral at best. Some parents found it daunting, describing it as “off-putting” and likening it to a school textbook in appearance (participants 4, 6 and 9):

“What put me off was the book itself because it looks like a textbook and the first thing you think of is oh my god I feel like a kid you know, I'm 40 years old. I really don't want to be doing this again” (participant 9)

Despite these reservations, “quick wins” in the first weeks boosted parents' confidence in the program and kept them motivated to take part. Parents reported particular success in this period with behavior charts, using descriptive praise and being consistent with ground rules.

“when I started I was at ground zero you know the, I was at rock bottom, I couldn't have got any lower so them few initial weeks are fantastic… you know it's just the tiny little things but just repeat, repeat, repeat I repeat the times out.. just little things and you get to a place where the child is responding” (participant 4).

##### Impact of the Program

Parents reported many positive impacts of the program, on their children, on themselves and more broadly on the wider family. Accounts indicated that by the end of the program, parents were more actively engaged with their children; more consistent in their parenting, and generally more structured in their approach and had a greater sense of control. As evidenced in the quantitative data, self-efficacy was very much improved and children's behavior was perceived to be less problematic overall. Parents took pride in the change that had been achieved and reported increases in their own wellbeing and mental-health. Reduced stress in relation to parenting was highlighted as a particular benefit and their improved mental health was attributed to this.

*Impact on the Parent:* Parents' accounts of the change within themselves were striking. The program had been transformative for all five who completed it. Not only were they all able to identify specific ways in which their parenting skills had improved, there were global statements that they were “better parents” and “changed people” as a result of the program. They were less stressed, calmer and happier in themselves and were looking forward with enjoyment to time and activities with their children.

“It has kept me calm, it was like being a new Mum again in a way… even though [X] is eight” (participant 5)

“I feel a lot more like a parent now than I was then” cause then I was just feeling embarrassed, low mood, really low, like I have no control over these children whatsoever what I do, now I feel I'm a better parent because of it (participant 8)

“The fact that I smile and laugh every day and I don't necessarily, I can't remember the last time I've asked my parents to have my kids overnight because I enjoy weekends” (participant 4)

“It's completely changed my whole world, 6 months ago is it, yeah summertime, I was in a terrible place with my mental health and it's just turned everything around it really has… having a strategy and a routine for the children and knowing how to handle their difficult behavior, it helps improve my wellbeing, knowing that I can cope, knowing that I can deal with it, erm if I get stuck I just refer to the book” (participant 6)

“It's made me feel a lot more confident in myself, knowing that these things can happen and that I can actually deal with them instead of trying to run away and hide which is what I used to do, bury my head in the sand like an ostrich” (participant 9).

*Impact on the Child:* Parents reported their children to be happier and more affectionate as a result of the changes parents has been able to make, describing a better child-parent relationship as well as noting significant improvements in behavior, which also transferred to other environments, including school. Two parents used the phrase “a new child”.

“[X] is a completely different child. I get hugs, I get kisses, temper tantrums are virtually gone erm, you know, the need for shouting or having to really deal with [X] in that way is not required (participant 4)

“The kids are a lot happier, they're a lot happier now, ‘cause I'm taking more time with them, sitting down and giving them this and some attention and I didn't know what I was doing before. I do now though, I'm glad I do it (participant 8)

Children were described as less oppositional overall and displayed a greater understanding of consequences. Parents recognized their own role in changing their own parenting behavior to achieve these outcomes and discussed improvements with pride. Communication between parents and children was also better and parents described themselves as feeling ‘closer' to their children. Parents reported enjoying time with their children and looking forward to their time together in marked contrast to their reflection of before the program. “I enjoy spending time with my children now, erm quality of life is just so different” (participant 4).

##### Broader Impacts

Parents reported additional impacts from the program relating to better communication within the wider family and more openness between all family members. Parents were more hopeful about their own lives and the future life chances of their children.

“[X] was going to grow up and at 13, he was going to be out, he was going to end up smoking weed or drinking or hanging round with the wrong crowd or I dread to think, but I think I'm going to have a lovely well rounded teenager and I'm not scared of that thought” (participant 4)

Participants with younger children also recognized the opportunity for them to benefit from the program: “all the things I've got in this book I didn't have when [X] and [X] were little so obviously it's going to be easier to put them into place for him because he's down here and not up here you know, I can teach him without the mistakes (participant 9).

#### Theme 2: Valuable Aspects and Suggestions for Improvement

Parents unanimously felt that the support they had been given to complete the program was invaluable. When asked whether they felt it was a necessary component of success all five felt that some face to face contact would be required for anyone completing the program in the future. Views on the possibility of group delivery were mixed and although some thought that the normalizing aspects of a group session with peers might be beneficial, reservations about sharing essentially private information with strangers meant this was not a viable option. It was clear that the one-to-one support they had received had ensured understanding of the book, and provided opportunity to discuss strategies:

“I think I'd have given up, I think by week 2, week 3 if I'd not seen a response on my own without having someone to talk to. Yes I would have just given up and it's awful to admit that but it's the truth” (participant 4)

“… sometimes I found myself slipping like I did have to actually look at the book and think “what should I do now” … it was good if like there was sometimes I didn't understand something or knowing how to approach a situation correctly … sometimes it's better to have that bit more input you know, am I doing it right?” (Participant 5)

Participants discounted entirely the (hypothetical) option of online delivery although there was some agreement that it could potentially be feasible if accompanied by effective telephone support, particularly at crisis points:

“It's a robot, it's a computer, it's not one to one, if you had a question it can't answer you” (participant 6)

“Doing it on a computer or tablet you're not going to stick with it, I wouldn't have, you need that interaction… that [online delivery with telephone contact] might be good if you know, you just sort of came on week one and there was maybe, like there's in mental health, a crisis team that you can ring” (participant 4)

Others felt that that it would be difficult to discuss parenting challenges on the telephone indicating the need for a rapport to be built first:

“nooooo… like things that we talk about well I would be like ‘is she pulling her face' or like ‘is she' cause like you can't see her you just don't know” (participant 5)

Parents were primarily recruited to the study via referral from adult mental health services and the independence of the study from children's services seemed to facilitate openness about parenting difficulties. Parents valued the normalizing aspects of the intervention, particularly discussion of the interaction between parenting stress and mental health and did not seem to experience their involvement as stigmatizing:

“A lot of parents clam up as soon as that starts to happen [struggling with children] because they're scared of letting someone like social services in… they're going to come in, they're going to sweep in, take your kids, bye bye, there goes your family” (participant 9)

“well this is the thing ‘cause I didn't know that this psychosis was very common and that you'd had people, clients like me, who were err suffering the same sort of thing but, it makes me happy that they will accept this is a mental illness and try and help you with it and still be a good Mum” (participant 8)

Parents made several practical suggestions for improvements to the workbook. They recommended a more aesthetically pleasing design to counter its textbook appearance and encourage better engagement. The need for a more inclusive book was highlighted, such as greater representation of more diverse family types, especially single parent families, and a larger font and simplified text.

Parents found some wording and phrases difficult to understand at first, for example ‘incidental teaching' and felt that some simplification was required but on the whole found the workbook clear and concise. Opportunities to discuss any sections that were difficult to follow were valued and showed the importance of having someone to discuss progress with:

“There were a couple of sections on 1 week that I just couldn't get my head round, I couldn't understand the phrasing, I couldn't get behind the concept and I struggled to deal sort of that week… I struggled to reach the full benefits but then I think when I saw [facilitator] to review the week she said don't get hung up on the things that you can't, just concentrate on the things that you do” (participant 4).

Participation in the case series involved weekly assessment of mental health and parents valued these discussions, which enabled the linking of mood and well-being to family stress and challenges. One parent recommended the monitoring of parental health in the workbook:

“Maybe something about your mental health and how you're going through the book and, and you know a graph or something or a place to erm, I don't know week one make a few notes or you know you, at the end of each chapter… a summary section for parents to write in of how they've thought that the week went and maybe a graph as well just so the parent could see because all about mental health, it's peak and troughs you know it's ups and downs, ups and downs and I think maybe if I could have seen you know erm, oh I've had a good week or on a bad week but I put a little note at the side what that blip was that maybe you know that, that could have been beneficial” (participant 4).

## Discussion

This is the first study to systematically explore the use of a guided self-help parenting intervention with parents experiencing psychosis. Recruitment rates were good and considerable change in outcomes was reported over time for the 50% who completed follow up. The remaining five completed only 40–60% of the intervention but it is noteworthy that all participants saw significant improvements across measures during the program. Very high acceptability ratings were reported and the qualitative evaluation was extremely positive. The practicality aspect of the intervention was rated with the lowest satisfaction for those ending prior to week 10.

Previous research had highlighted the challenges of recruiting parents and service users into research and retaining participants in parenting interventions, therefore weekly guidance and assistance was offered. This was largely due to low levels of confidence, poor literacy skills, cognitive deficits and motivation. Initial barriers to progress and engagement were described as feeling overwhelmed and a mistrust of services. Two participants exited the study early due to struggling with literacy and chaotic environments including house moves. Support to understand and implement strategies and exercises in the workbook were essential. This improved as perceptions of self and parent-child relationship shifted throughout the course of the intervention.

Clinically significant reductions (>25%) across mental health and parent-child outcome measures were demonstrated on weekly measures. The magnitude of change was significant across mental health and parenting practices, parental confidence, and child behavior outcome measures, except for PANSS. The frequency, severity and levels of distress caused by hallucinations and delusions on PSYRATS was significantly reduced during the ten weeks of the intervention and continued to improve post-intervention and at follow-up. At completion, all participants were no longer meeting criteria for ‘clinical' categories on outcome measures. Similarly, improvements across facets of child disruptive behaviors, positive parenting practices and parental self-efficacy was comparable to previous research (21, 25, 28, 31). Participants moved from the “clinical” category showing fewer child behavior problems and less parental distress on the ECBI and were within the “normal” range for parenting practices on the PS. Parental self-efficacy showed a substantial increase on the PTC. Improvements were maintained at follow-up, except for depression, anxiety and stress scores on the DASS-21. Where significant effects were found, effect sizes were very large (above 1.0).

The qualitative evaluation revealed that parents who completed the full 10 weeks of the intervention were extremely positive about its impacts, despite initial reservations and doubts about its ability to effect change. Their accounts also gave insights into the mechanisms of change by which parental mental health and wellbeing were improved. Parents described greater self-efficacy in their parenting as a result of the intervention. A more positive approach to parenting and improved behavior management strategies combined to improve child behavior. Parents spoke of enjoying time with their children rather than finding interactions stressful. Stress reactivity relating to psychosis has been demonstrated in numerous studies (54) and stress has been shown to worsen psychiatric symptoms in people with psychosis. It is also well established that high conflict family environments are linked to greater symptoms and increased likelihood of relapse in schizophrenia (55). In addition, there is evidence that more severe symptoms are associated with higher levels of parenting stress in people with serious mental illness and, conversely, that parenting improves when symptoms decline (56). Hence, it seems likely that parental wellbeing is enhanced by the stress of parenting being reduced. The accounts of the five participants who did not fully complete the intervention are missing of course, and it may be assumed that a different picture may well have emerged had they been included. Nonetheless, the finding that 50% of those who started the program experienced such significant change is important, and indicates that with the required adjustments to enable parents to engage with such an intervention, significant positive impacts are possible.

### Strengths and Limitations

No participants had previously taken part in research and all ten presented with chronic symptomology. Therefore, the process of change could be conceptualized differently than those within other services presenting with acute as opposed to chronic mental health challenges. Despite aiming to recruit both mothers and fathers, no fathers took part. The majority of these mothers were also single, experiencing socioeconomic disadvantage and facing adversity. Although this reflects the typical household makeup of children living with a parent with psychosis (57), future research should seek to include fathers and parents from broader socioeconomic backgrounds where possible. The majority of participants were also White British. Spoken and written English was an inclusion criterion that will have precluded some groups of parents from taking part (for example immigrants). Suitability of the intervention for more diverse populations would need to be established in a larger trial.

With parents acting as their own baseline controls, the extent to which change can be attributed to the intervention cannot be fully established. To counteract this limitation, any significant lifestyle, family or medication changes were monitored. No significant changes were reported. Case series methodology is restrictive in its ability to demonstrate treatment efficacy; however, applying a multiple baseline design, session-by-session measures and reporting effect sizes, strengthens the findings. Autocorrelation of data was not assessed and controlled for which may have increased the likelihood of type I error.

The same researcher completed all assessments from baseline to follow-up which could cause biases in design and interpretation of outcomes. Although developing a relationship over time with the participants was a strength, to mitigate potential interpretation biases, self-report measures were used and a subset of mental health interviews were listened to by the wider research team. Follow-up at 3 and 6 months showed that gains acquired during the intervention were largely durable; however, this length of follow was insufficient to determine whether improvements can be maintained in the longer term. A longer follow up period, ideally a year or more, would offer insight into the strategies, knowledge and techniques that remain useful and implemented by families in the longer term.

### Conclusion and Future Directions

Preliminary indications arising from this study are that a home-visiting parenting intervention for parents experiencing psychosis could be feasible, effective and valuable. Further studies involving larger and more diverse samples and a randomized controlled design are needed to substantiate these outcomes and more work surrounding successful implementation is needed.

Establishing the impact of parenting interventions for parents with different levels of need, varied illness length and the impact on quality of life will ensure support can be targeted and appropriate. It is also essential to identify the active ingredients within a parenting intervention that drive or prevent change (34).

The optimum modality and duration of parenting interventions needs to be adequately examined to ascertain the most beneficial method. Feedback from parents suggested that less repetition and using audio-guidance or visual supports such as video animations or infographics could assist those with poor literacy. This could also address some of the practical challenges reported. Utilizing technology (for example, smartphone applications and electronic behavior diaries) could also be of use to self-monitor behavior change. There are clear benefits from simply being listened to, having distress recognized and receiving warmth. Exploring the role of face-to-face support will disentangle intervention benefits from the benefits of modality type. Staff engagement and awareness of the dual demands of parenting when experiencing a serious mental health challenge needs to be of focus to ensure efficacious parenting interventions are no longer under-utilized in mainstream services. Examination of the most appropriate and useful ways of disseminating and integrating future work into services with a multi-agency approach in mind is essential to target hard-to-reach families.

This study has established that the use of the Triple-P Self-Help Workbook, using a guided and supportive framework, is feasible to deliver to parents experiencing psychosis. Positive outcomes were apparent across mental health measures with some participants no longer experiencing delusions or hallucinations and others moving out of clinical ranges. Child behavior, parenting practices and parent-child interactions all improved for each participant completing the intervention highlighting its potential promise as an intervention. Future development work with a focus on implementation should seek to increase the acceptability of the intervention to ensure completion and increase retention to follow up so that its effects may be determined more robustly.

## Data Availability Statement

The raw data supporting the conclusions of this article will be made available by the authors, without undue reservation.

## Ethics Statement

The studies involving human participants were reviewed and approved by Greater Manchester West National Research Ethics Committee. The patients/participants provided their written informed consent to participate in this study.

## Author Contributions

LW: conceptualization, designing the study, data collection, quantitative data analysis, and writing the manuscript. RC: conceptualization, designing the study, supervision, and reviewing and editing the manuscript. RD: conceptualization, designing the study, and supervision LG: conceptualization, designing the study, supervision, qualitative data analysis, and writing, reviewing and editing the manuscript. All authors contributed to the article and approved the submitted version.

## Funding

LW was supported by a President's Doctoral Scholar award from The University of Manchester.

## Conflict of Interest

The authors declare that the research was conducted in the absence of any commercial or financial relationships that could be construed as a potential conflict of interest.

## Publisher's Note

All claims expressed in this article are solely those of the authors and do not necessarily represent those of their affiliated organizations, or those of the publisher, the editors and the reviewers. Any product that may be evaluated in this article, or claim that may be made by its manufacturer, is not guaranteed or endorsed by the publisher.
